# Disentangling pharmacological and expectation effects in antidepressant discontinuation among patients with fully remitted major depressive disorder: study protocol of a randomized, open-hidden discontinuation trial

**DOI:** 10.1186/s12888-023-04941-3

**Published:** 2023-06-21

**Authors:** Carina Meißner, Claire Warren, Tahmine Fadai, Amke Müller, Antonia Zapf, Susanne Lezius, Ann-Kathrin Ozga, Irina Falkenberg, Tilo Kircher, Yvonne Nestoriuc

**Affiliations:** 1 Clinical Psychology, Helmut-Schmidt-University/University of the Federal Armed Forces Hamburg, Holstenhofweg 85, 22043 Hamburg, Germany; 2 Institute of Systems Neuroscience, University-Medical Center Hamburg-Eppendorf, Martinistraße 52, 20246 Hamburg, Germany; 3 Institute of Medical Biometry and Epidemiology, University Medical Center Hamburg-Eppendorf, Hamburg, Germany; 4 Department of Psychiatry, University of Marburg, Marburg, Germany

**Keywords:** Depressive disorder, Psychotropic drugs, Drug tapering, Treatment expectation, Nocebo effect

## Abstract

**Background:**

Antidepressants are established as an evidence-based, guideline-recommended treatment for Major Depressive Disorder. Prescriptions have markedly increased in past decades, with a specific surge in maintenance prescribing. Patients often remain on antidepressants longer than clinically necessary. When attempting to stop, many patients experience adverse discontinuation symptoms. Discontinuation symptoms can be debilitating and hinder successful discontinuation. While discontinuation symptoms can result from pharmacological effects, evidence on nocebo-induced side effects of antidepressant use suggests that patients' expectations may also influence occurrence.

**Methods:**

To disentangle pharmacological and expectation effects in antidepressant discontinuation, patients with fully remitted Major Depressive Disorder who fulfill German guideline recommendations to discontinue will either remain on or discontinue their antidepressant. Participants' expectations will be manipulated by varying verbal instructions using an open-hidden paradigm. Within the open trial arms, participants will receive full information about treatment, i.e., high expectation. Within the hidden trial arms, participants will be informed about a 50% chance of discontinuing versus remaining on their antidepressant, i.e., moderate expectation. A total of* N* = 196 participants will be randomly assigned to either of the four experimental groups: open discontinuation (OD; *n* = 49), hidden discontinuation (HD; *n* = 49), open continuation (OC; *n* = 49), or hidden continuation (HC; *n* = 49). Discontinuation symptom load during the 13-week experimental phase will be our primary outcome measure. Secondary outcome measures include discontinuation symptom load during the subsequent 39-week clinical observation phase, recurrence during the 13-week experimental period, recurrence over the course of the complete 52-week trial evaluated in a time-to-event analysis, and stress, anxiety, and participants’ attentional and emotional processing at 13 weeks post-baseline. Blood and saliva samples will be taken as objective markers of antidepressant blood serum level and stress. Optional rsfMRI measurements will be scheduled.

**Discussion:**

Until today, no study has explored the interplay of pharmacological effects and patients’ expectations during antidepressant discontinuation. Disentangling their effects has important implications for understanding mechanisms underlying adverse discontinuation symptoms. Results can inform strategies to manage discontinuation symptoms and optimize expectations in order to help patients and physicians discontinue antidepressants more safely and effectively.

**Trial registration:**

ClinicalTrials.gov (NCT05191277), January 13, 2022.

**Supplementary Information:**

The online version contains supplementary material available at 10.1186/s12888-023-04941-3.

## Background

Antidepressants are established as an evidence-based, guideline-recommended treatment for moderate to severe episodes of Major Depressive Disorder (MDD; [1–3]). Prescription rates have increased markedly in high-income countries over recent decades, with a specific surge in maintenance prescribing [1, 4–6]. Maintenance treatment with antidepressants following sustained remission is thought to reduce the risk of recurrence [7]. Treatment guidelines recommend maintenance treatment for several months in case of a single episode, and two years or longer for recurrent episodes [8–11]. However, prophylactic effects of maintenance treatment have rarely been studied in trials of more than 52 weeks. To date, consensus on duration and effectiveness is lacking [9, 12, 13].

Prescriptions of antidepressants have increased by more than 30% over the last decade, primarily due to selective serotonin reuptake inhibitors (SSRIs) and selective serotonin-norepinephrine reuptake inhibitors (SNRIs) [3]. Increased maintenance prescribing burdens healthcare costs [1, 3] and is associated with individual risks. Adverse side effects of antidepressant use include sexual dysfunction, sedation, agitation, emotional difficulties, gastrointestinal problems, weight gain, orthostatic dysregulation, or QT interval prolongation and subsequent risk of cardiac arrhythmias [14–16]. Side effects often persist during long-term use [17] and motivate patients’ wish to discontinue [18–22].

As 30 to 50% of maintenance treatment lacks clinical indication, a significant proportion of patients with antidepressant use may consider discontinuation [13, 23, 24]. Yet, with no safe and effective discontinuation rationale established, these patients face multiple challenges [25–28]: Patients are not routinely informed about timelines and methods for discontinuation, leading to abrupt or unsupervised attempts, unsafe tapering regimens, or refraining from discontinuation at all [21, 29, 30]. Patients are not regularly reviewed by clinicians, and patients and clinicians often perceive the opposite as responsible for discussing discontinuation [31–33]. Fear of adverse discontinuation symptoms or recurrence following discontinuation constitute further barriers [21, 34].

Discontinuation symptoms are common and multifaceted [25, 35, 36]. Symptoms include hyperarousal, gastrointestinal problems, flu-like syndromes, sensory disturbances such as brain and body zaps, or insomnia [35, 36]. Incidence ranges from 27 to 86% of patients affected, with a systematic review reporting a weighted average of 56% [25]. Up to half of patients with discontinuation symptoms classified these as severe [25]. Symptom persistence varies from weeks to months, with up to a quarter of patients reporting discontinuation symptoms lasting longer than six weeks [25, 37–39]. Reversal of dose reduction may alleviate symptoms [26, 37], but contributes to non-indicated long-term use.

Certain discontinuation symptoms, such as anxiety, irritability, or suicidal thoughts, resemble depressive symptoms. This resemblance often confounds assessment of recurrence in discontinuation trials [28]. Nevertheless, some patients appear to be at increased risk for recurrence following discontinuation [7, 40]. Clinical and demographic variables were found to be of limited use as indicators of recurrence risk [41, 42]. A recent imaging study by Berwian et al. [43] identified changes in connectivity between dorsolateral prefrontal cortex and posterior default mode network as a potential predictor, but remains to be replicated. Validated predictors of recurrence risk have not yet been established and differential diagnosis between recurrence and discontinuation symptoms remains challenging. This gap in knowledge presents a risk for inadequate treatment decisions concerning antidepressant discontinuation versus continued used [37, 44].

Antidepressant discontinuation is associated with various expectations [21, 45]. Patients may expect to get rid of antidepressant-related side effects, but also expect recurrence of depressive symptoms or discontinuation symptoms. Patients’ negative expectations concerning their health state can result in adverse health outcomes via the nocebo effect [46]. Evidence of expectation effects on antidepressant efficacy and tolerability [47–50], i.e., reduced depressive symptoms [50] and nocebo-induced adverse side effects [49] under placebo conditions, indicates that expectations also influence antidepressant discontinuation [45]. Negative expectations towards discontinuation can result from prior negative discontinuation experiences [21] or from negative reports within the social environment, including social media and online platforms [51, 52]. Verbal information provided by the prescribing physician on MDD illness framework or antidepressant mode of action can also induce negative expectations [21]. Patients who internalized a chemical imbalance model of depression are likely to believe that discontinuation of their antidepressant will re-establish the chemical imbalance, leading to recurrence [53]. In order to understand the roles of pharmacology and expectation in antidepressant discontinuation, experimental studies that systematically modulate pharmacological and expectation effects, while carefully distinguishing depressive from discontinuation symptoms, are needed.

### Aim and hypotheses

The primary aim of our trial is to disentangle how pharmacological and expectation effects contribute to discontinuation symptom load in antidepressant discontinuation. We hypothesize that treatment (discontinuation vs. continuation) and treatment expectation (high vs. moderate) interact in modulating discontinuation symptom load among patients. If we find a significant interaction effect of treatment and treatment expectation, we expect that i) patients who remain on their antidepressant will show a higher discontinuation symptom load with moderate than with high expectation, ii) patients with moderate treatment expectation will show a higher discontinuation symptom load if the antidepressant is discontinued versus continued, and iii) patients who discontinue their antidepressant will show a higher discontinuation symptom load with high than with moderate treatment expectation. We assume that the relationship between treatment expectation and discontinuation symptom load will vary according to stress ratings, antidepressant-related side effects, prior discontinuation experience, neuroticism, anxiety, somatosensory amplification, and illness framework.

## Methods

### Study design

**Fig. 1:**
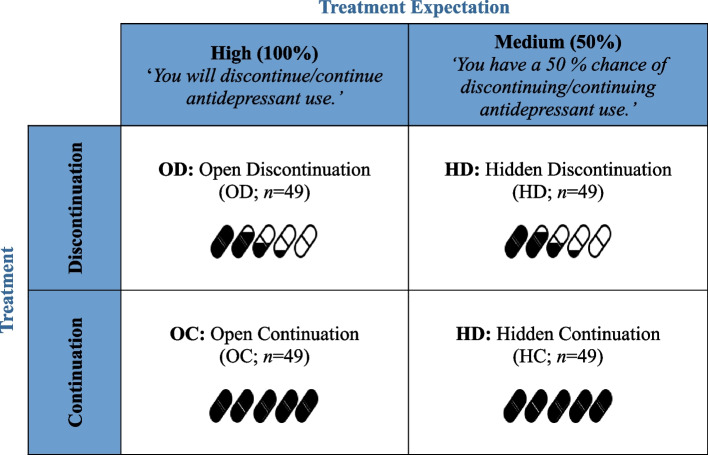
Randomized, balanced open-hidden discontinuation design

This prospective, randomized, parallel-group, partly blinded, open-hidden discontinuation trial with a 2 × 2-factorial design will systematically vary the factors *Treatment* and *Expectation* to investigate the interaction of pharmacological and expectation effects in modulating discontinuation symptom load. 196 patients with fully remitted MDD and an indication to discontinue antidepressant use will be randomly allocated to open discontinuation (OD), hidden discontinuation (HD), open continuation (OC), or hidden continuation (HC) of their antidepressant (Fig. 1). Our trial is part of a collaborative research center (CRC; TRR 289 Treatment Expectation: treatment-expectation.de/en/).

### Interventions

#### Treatment

Treatment will be manipulated as discontinuation versus continuation of antidepressant use. At the beginning of the 13-week experimental phase, all participants will remain on their prescribed antidepressant and initial dose, though newly encapsulated, for a 1-week run-in phase to control for tablet appearance effects. Run-in will be followed by a 4-week tapering/continuation phase plus 8-week monitoring. During the 4-week tapering phase, discontinuation groups OD and HD will receive encapsulated tablets with decreasing doses of their prescribed antidepressant. Pre-specified dose-reduction schemes determine that i) doses will be gradually reduced over (at least) 4 weeks in at least 5 dose reduction steps, ii) dose reduction steps become smaller over the discontinuation process, and iii) overall dose reduction will be higher in the first two weeks than in the last two weeks. This tapering regimen approximates the hyperbolic discontinuation method advocated by recent research [54]. Continuation groups OC and HC will receive encapsulated tablets containing initially prescribed doses of prescribed antidepressant. During the 8-week monitoring phase, participants in the open trial arms will receive encapsulated tablets as open medication and open-label placebo, respectively. Participants in the hidden trial arms will receive encapsulated tablets of medication or placebo, respectively, and remain blinded to treatment.

#### Expectation

Expectation will be manipulated by varying verbal instructions using the open-hidden paradigm (high vs. moderate). Participants in the open trial arms will receive full information about treatment, i.e., high expectation. Participants in the hidden trial arms will receive information about a 50% chance of either discontinuing or continuing antidepressant use, i.e., moderate expectation. All tablets for all participants will look identical throughout the experimental phase to keep both participants and study staff unaware of group assignment within the hidden trial arms.

### Randomization

A block randomization with varied block sizes will be performed externally by the Medical Biometry department of the University Medical Center Hamburg-Eppendorf, Hamburg, Germany. Block size will be unknown to the study staff. Participants will be allocated 1:1:1:1 to OD, HD, OC, and HC groups. Randomization will be stratified by duration of antidepressant use, with 24 months as a marker for long-term use (< 24 months vs. > 24 months, 3:7), and antidepressant-associated risk of developing discontinuation symptoms (moderate [citalopram, escitalopram, sertraline, duloxetine] vs. higher or unknown risk [paroxetine, venlafaxine, mirtazapine], 1:1).

### Blinding

Digital randomization lists will contain allocation sequences and will be passed on to unblinded randomization officers who have no personal contact with participants to assign participants to interventions. In case of assignment to one of the double-blinded hidden trial arms, both participants and study staff will be blinded to group assignment. Participants in the hidden trial arms, their prescribing physicians, and study staff will be debriefed 13 weeks post-baseline at t9. Staff responsible for data analysis will be blinded to group assignment. Digital and paper key lists containing pseudonym, name, contact details, experimental group, medication, and current dosage will be prepared to ensure emergency unblinding (e.g., in case of hospitalization).

### Participants and recruitment

Participants will be recruited from the psychiatric outpatient clinics of the University Medical Center Hamburg-Eppendorf, Hamburg, Germany. The study will be advertised via support groups, psychiatric practices, general practitioners, and pharmacies. Digital and analogue methods such as media and newspaper articles, advertisement in public transport, social media, online forums, and leaflets will be used. Interested patients can contact the study team for further study information and a first screening telephone interview (S1). Potential participants will be invited for an on-site screening interview (S2), consisting of an in-depth clinical interview to assess eligibility. Indication for discontinuation will be assessed in accordance with German guideline recommendations [26] and in consultation with the prescribing physician. Detailed eligibility criteria are summarized in Table 1.

**Table 1:** Inclusion and exclusion criteria

Inclusion criteria
1. Adult patients (18–75 years) with fully remitted MDD, single or recurrent, as main diagnosis, confirmed by prescribing physician and SCID-5-CV [55, 56]
2. Use of SSRI/SNRI (citalopram: 20-40 mg, escitalopram: 10-20 mg, sertraline: 75-150 mg, venlafaxine: 75-150 mg, duloxetine: 60-100 mg, paroxetine: 20-40 mg) or NaSSA (mirtazapine: 30-45 mg)
3. Discontinuation wish by patient, supported by prescribing physician
4. Fulfilment of guideline recommendations to discontinue antidepressant use [26]: a) response to antidepressant, b) symptom remission for at least four months (first episode)/ 2 years (2 or more episodes with significant functional impairment) and c) concurrent use of antidepressant medication (at least 4 weeks on a steady dose)
Exclusion criteria
1. Acute or chronic somatic illness and/or use of medication which might interfere with depressive disorder, antidepressant use, or proposed study
2. Acute suicidality, psychotic symptoms, substance abuse or addiction within the last 12 months, current mania or hypomania confirmed by SCID-5-CV, or other psychopathology which might interfere with depressive disorder, AM, or proposed study
3. Any history of bipolar disorder or psychosis, confirmed by SCID-5-CV
4. Severe stressful life events (e.g., death of a family member) within six months prior to study participation
5. Insufficient German language proficiency
6. No informed consent
7. MRI-specific exclusion criteria, if applicable: phobic anxiety, claustrophobia, ferromagnetic implants, etc

### Sample size calculation

The target sample size of *N* = 168 (*n* = 42 in each experimental group) is based on medium to large effect sizes for expectation effects on depressive symptoms and treatment outcome [47, 50] and hypothesized small to medium interaction effects with pharmacological modulations. A medium-sized effect (*f* = 0.22) for the interaction of expectation and pharmacological modulation was assumed in an ANOVA (with main effects and interactions) with a between-within interaction using a power of 80% and two-sided significance level of 0.05. Including oversampling with an anticipated dropout rate of 15%, our recruitment target is *N* = 196 (with *n* = 49 per group). Sample size calculation was conducted using G*Power (version 3.1.9.2; [57]).

### Procedure

**Fig. 2:**
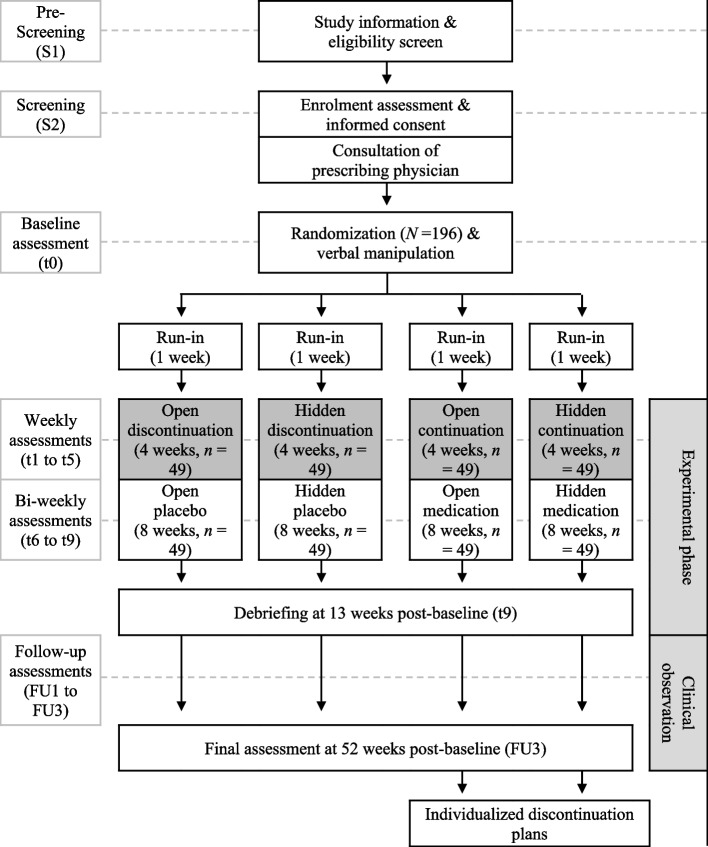
Participant flow through the trial

Assessments will be conducted according to pre-defined standard operating procedures (SOPs) at the Institute of Systems Neuroscience, University-Medical Center Hamburg-Eppendorf, Hamburg, Germany. Figure 2 shows participant flow through the trial. A study physician or psychologist will ask eligible patients to give written informed consent for study participation, blood analysis, optional saliva sampling, and optional rsfMRI assessment. A release from the medical confidentiality obligation of the prescribing physician will be obtained. Prescribing physicians will be asked to confirm absence of objection against discontinuation, MDD as primary diagnosis for initial antidepressant prescription, current type and dosage of antidepressant, duration of antidepressant use, medication switches, and frequency of visits. Participants will then be randomized, invited for baseline assessment t0, and informed about group allocation.

Assessments during the 13-week experimental phase, i.e., weekly from t0 to t5 and biweekly from t5 to t9, will be interview- and questionnaire-based. Each study visit begins with a clinical interview to assess depressive symptoms, adverse events (AEs), adherence, and clinical impression. Participants will then be asked to fill digital questionnaire batteries programmed with LimeSurvey [58]. A study physician or psychologist will examine safety-relevant data and, if necessary, discuss these data immediately with the participant. Otherwise, the study visit will end with handing out the study medication. Saliva samples will be taken between t0 and t1, blood samples at t1 and t9, and rsfMRI measurements at t0 or t1. At t9, an emotional interference paradigm will be administered.

Following the 13-week experimental phase, participants will enter a 39-week clinical observation phase. The clinical observation phase consists of phone or online calls and online-questionnaires at FU1-3, i.e., 26, 39, and 52 weeks post-baseline. At FU3, participants who continued antidepressant use will be offered an individualized discontinuation plan. All participants will receive a reimbursement of €150 for full participation in the study, or pro rata reimbursement in case of incomplete participation. There will be two payouts of €75, one following completion of the experimental phase and another following completion of the trial. In case of participation in rsfMRI measurement, participants will receive an additional €30.

### Primary outcome

Our primary outcome *discontinuation symptom load* over the course of the experimental phase will be assessed with the *Discontinuation Emergent Signs and Symptoms Scale* (DESS; [59]). The DESS is a self-report measure incorporating 43 symptoms of antidepressant discontinuation. Participants will rate intensity of each discontinuation symptom on a 4-point response-scale (0 *‘not present’*, 1 *‘mild’*, 2 *‘moderate’*, 3 *‘severe’*; [60]). Sum scores range between 0–129. Higher scores indicate more pronounced discontinuation symptoms. Discontinuation symptom load will be measured as area under the curve (AUC) based on assessments from t2 until t9, adjusted for baseline symptoms reported at t1. Table 2 provides an overview of assessments according to SPIRIT guidelines [61].

**Table 2:** Schedule of enrolment, interventions, and assessments according to SPIRIT-PRO

	**STUDY PERIOD**								
	**Enrolment**		**Allocation**	**Experimental phase**			**Clinical observation**		
**Timepoint**	**S1**	**S2**	**t0**	**t1-t5**	**t6-t8**	**t9**	**FU1**	**FU2**	**FU3**
**Weeks**	**-2**	**-1**	**0**	**1/2/3/4/5**	**7/9/11**	**13**	**26**	**39**	**52**
**Enrolment**									
Eligibility screen	✓	✓							
Informed consent		✓							
Initial consultation prescribing physician		✓							
Randomization			✓						
**Interventions**									
Discontinuation versus continuation of antidepressant				✓	✓	✓			
High versus moderate expectation (open versus hidden treatment)				✓	✓	✓			
**Assessment**									
**Primary outcome measures**									
Discontinuation symptom load (DESS)			✓	✓	✓	✓			
**Secondary outcome measures**									
Discontinuation symptom load (DESS)							✓	✓	✓
Recurrence during experimental phase				✓	✓	✓			
Recurrence over the course of the trial				✓	✓	✓	✓	✓	✓
Change in psychophysiological stress (PSS-10) ^a^			✓			✓			
Change in state anxiety (STADI State) ^a^			✓			✓			
Attentional, affective processing (Posner task) ^a^						✓			
**Possible modulators**									
Psychophysiological stress (PSS-10) ^a^			✓						
Side effects of antidepressant medication (GASE) ^a^			✓			✓			
Prior discontinuation experience (GEEE_PRE_) ^a^			✓						
Neuroticism (BFI-10) ^a^			✓						
Trait anxiety (STADI Trait) ^a^			✓						
Somatosensory amplification (SSAS) ^a^			✓						
Illness perception (single item)			✓						
**Further assessments**									
Optional: rsfMRI			✓	✓ (t1)					
Antidepressant blood serum level				✓(t1)		✓			
Trait marker stress ^a^			✓						
Adherence (single item)			✓	✓	✓	✓	✓	✓	✓
Self-reported depressive symptoms (BDI-II) ^a^		✓	✓	✓	✓	✓	✓	✓	✓
Expert-rated depressive symptoms (MADRS) ^a^		✓	✓	✓	✓	✓	✓	✓	✓
Prior discontinuation symptoms (DESS_PAST_)			✓						
Current treatment effects (GEEE_ACT_) ^a^			✓	✓	✓	✓	✓	✓	✓
Expectations (TEX-Q)^ a^			✓	✓	✓	✓			
Expectations (GEEE_EXP_) ^a^			✓	✓	✓	✓	✓	✓	✓
Behavioral inhibition/approach (BIS-BAS scale) ^a^			✓						
Psychopathology (SCID-5-CV interview) ^a^		✓							
Personality traits (BFI-10) ^a^			✓						
Well-being (SWEMWBS)			✓	✓	✓	✓	✓	✓	✓
Depression and anxiety (PHQ-4)							✓	✓	✓
Subjective impairment (PDI) ^a^			✓	✓(t1,t5)		✓			
Substance use ^a^				✓(t1,t5)	✓(t7)	✓			
Warmth & competence ^a^			✓	✓(t5)		✓			
Adverse events (single safety items)			✓	✓	✓	✓	✓	✓	✓
Suspicions about treatment (GEEE_END_) ^a^						✓			
Demographic ^a^ & medical characteristics			✓			✓	✓	✓	✓
**Debriefing & close-out**									
Debriefing						✓			
Consultation with prescribing physician						✓			
Individualized discontinuation plans for continuation groups									✓

*Note.* *S1* = pre-screening; *S2* = enrolment; *t* = assessment timepoint; *FU* = follow-up; *DESS* = Discontinuation Emergent Signs and Symptoms Scale; *PSS-10* = Perceived Stress Scale, 10 item version; *GASE* = Generic Assessment of Side Effects; *GEEE*_*PRE*_ = Generic Rating for Treatment Pre-Experiences, Treatment Expectations, and Treatment Effects (previous experiences); *BFI-10* = 10-item Big-5 Inventory; *STADI* = State-Trait-Anxiety-Depression-Scale; *SSAS* = Somatosensory Amplification Scale; *rsfMRI* = resting-state functional Magnetic Resonance Imaging; *BDI-II* = Beck-Depression-Inventory II; *MADRS* = Montgomery-Asberg Depression Rating Scale; *DESS*_*PAST*_ = Discontinuation Emergent Signs and Symptoms Scale (previous experiences); *GEEE*_*ACT *_= Generic Rating for Treatment Pre-Experiences, Treatment Expectations, and Treatment Effects (treatment effects); *TEX-Q* = Treatment Expectation Questionnaire, 15 item version; *GEEE*_*EXP*_ = Generic Rating for Treatment Pre-Experiences, Treatment Expectations, and Treatment Effects (treatment expectations); *BIS-BAS* scale = Behavioral Inhibition and Approach System Scale; *SCID-5-CV* = Structured Clinical Interview for DSM-5; *SWEMWBS* = Short Warwick-Edinburgh Mental Well-Being Scale; *PHQ-4* = Patient-Health-Questionnaire-4; *PDI* = Pain Disability Index (adapted to discontinuation symptoms); *GEEE*_*END*_ = Generic Rating for Treatment Pre-Experiences, Treatment Expectations, and Treatment Effects (suspicions about treatment).

^a^ Part of standardized psychometric test battery within our CRC

### Secondary outcomes

Secondary outcomes will include i) discontinuation symptom load over the clinical observation period, ii) recurrence over the experimental period, iii) recurrence over the course of the complete trial evaluated in a time-to-event analysis, differences in iv) stress and v) state anxiety from baseline to end of the experimental phase, and vi) attentional and emotional processing at the end of the experimental phase. *Discontinuation symptom load over the clinical observation period* will be based on DESS assessments from t9 until FU3 [59] to cover reported symptoms between week 13 until week 52 and measured as AUC. Both *recurrence over the experimental period* (t1-t9) and *recurrence over the course of the trial* (t1-FU3, evaluated in a time-to-event analysis) will be monitored via expert-rating and self-report measure. Recurrence will be defined as appearance of a new depressive episode after full, sustained remission of depressive symptoms. Study physicians and psychologists will use the *Montgomery-Asberg Depression Rating Scale* (MADRS) to assess depressive symptom severity by ten items with seven intensity ratings (0–6) each [62, 63]. Sum scores range between 0–60. Higher scores indicate more pronounced depressive symptoms. To standardize assessments, a German translation of the Structured Interview Guide for the Montgomery-Asberg Depression Rating Scale (SIGMA) will be used [64]. Participants will assess depressive symptoms with the *Beck Depression Inventory* (BDI-II; [65, 66]), a self-report measure that includes 21 items with 4 response options (0–3). Sum scores range between 0–63. Higher scores indicate more pronounced depressive symptoms. If recurrence is suspected, as indicated by BDI-II score > 19 or MADRS score > 21 over two consecutive study visits, corresponding sections of the expert-rated *Structured Clinical Interview for DSM-5 – Clinician Version* (SCID-5-CV; [56]) will be conducted to (dis-)confirm recurrence. In case of recurrence, beginning of the new depressive episode will be examined and recorded as calendar week. The *Perceived Stress Scale* (PSS-10; [67, 68]) will be used to assess *stress*. The PSS-10 includes ten items with five response options (0 *‘none of the time’* to 4 *‘very often’*). Sum scores range between 0–40. Difference scores will be calculated by subtracting scores at t1 from scores at t9 and range between -40 to 40. Higher scores indicate increased stress. The *State-Trait-Anxiety-Depression-Inventory* (STADI; [69, 70]) will be used to assess *state anxiety*. Two anxiety-related state subscales include five statements with four response options (1–4) each. State anxiety scores base on sum scores of both state anxiety subscales and range between 10–40. Difference scores will be calculated by subtracting scores at t1 from scores at t9, resulting in a range between -30 to 30. Higher scores indicate increased state anxiety.

We will use a modified emotional Posner task to assess *attentional and emotional processing* at t9. The Posner task manipulates attentional resources and provokes emotional responses using facial stimuli, activating limbic, prefrontal, and visuo-spatial brain circuits [71]. In short, participants respond as fast as possible to a dot target by button pressing, while neutral, happy, sad, or fearful faces are presented as distractors. Targets are preceded by either spatially-directing cues leading to covert shifts in the attentional focus (i.e., low attentional resources to process distractors) or non-spatial cues, leaving the attentional focus on the faces. We will measure reaction times in milliseconds (ms) for each condition and calculate difference scores for reaction times to happy - neutral faces, sad - neutral faces, and fearful - neutral faces under high attention to faces.

### Modulators

Stress, antidepressant-related side effects, prior discontinuation experience, neuroticism, trait anxiety, somatosensory amplification, and illness framework will be explored as modulators. *Stress* will be rated with the PSS-10 (see secondary outcomes). Antidepressant-related *side effects* will be measured with the *Generic Assessment of Side Effects Scale* (GASE, [72]). The GASE is a self-report measure including 36 symptom descriptions with 4 severity ratings (0 *‘not present’*, 1 *‘mild’*, 2 *‘moderate’*, 3 *‘severe’*). For every reported symptom, participants will indicate severity and whether they perceive this side effect as antidepressant-related. Sum scores of antidepressant-related symptom ratings range between 0–108. Higher scores indicate more pronounced antidepressant-related side effects. *Prior discontinuation experience* will be assessed with a modified version of the *Generic Rating Scale for Treatment Pre-Experiences* (GEEE_PRE_, [73]). Participants will indicate improvement (0 *‘no improvement’*—10 *‘greatest improvement imaginable’*) and worsening (0 *‘no worsening’*—10 *‘greatest worsening imaginable’*) of general condition attributed to the most recent discontinuation attempt on numeric rating scales (NRS). Difference scores will be calculated by subtracting worsening scores from improvement scores, resulting in a range between -10 to 10. Lower scores indicate a more negative discontinuation experience. Participants with no prior discontinuation experience will be assigned a value of 0. *Neuroticism* will be assessed with the Emotional Stability subscale of the *Brief Big Five Inventory* (BFI-10; [74]). The Emotional Stability subscale includes two items rated on a five-point scale (1 *‘disagree strongly’*—5 *‘agree strongly’*). Sum scores range between 2–10, with higher scores indicating higher neuroticism. The *State-Trait-Anxiety-Depression-Inventory* (STADI; 69, 70) will be used to assess *trait anxiety*. Two anxiety-related trait subscales include 5 statements with four response options (1–4) each. Trait anxiety scores base on sum scores of both trait anxiety subscales and range between 10–40. Higher scores indicate higher trait anxiety. The *Somatosensory Amplification Scale* (SSAS; [75, 76]) will be used to assess the amount of *somatosensory amplification*. Ten items will be rated on a five-point scale (1 ‘*not at all true’*—5 *‘extremely true’*). Sum scores range from 10–50, with higher scores indicating higher symptom amplification. Finally, participants will be asked about their *illness rationale*. A single item will be used to assess whether participants perceive MDD as a more biologically or psychologically caused disorder (0 *‘biologically caused’*—10 *‘psychologically caused’*). A higher score indicates an inclination towards psychological causes underlying MDD.

### Adherence

During the experimental phase, *treatment adherence* to study medication will be assessed in two ways. First, participants will indicate the number of days the study medication was taken since the last study visit. Second, study staff will assess treatment adherence via blood analysis at t1 and t9. Blood samples will be collected using white tubes without gel and will be analyzed with Liquid Chromatography Mass Spectrometry (LC–MS/MS) in accordance with EU guidelines as stated in the In-vitro-Diagnostic Device Regulation (IVDR; [77]). Storage and analysis will be conducted at the Department of Legal Medicine, University Medical Center Hamburg Eppendorf, Hamburg, Germany. During the clinical observation phase, participants will indicate whether they remained on or off antidepressants. In case of non-adherence, possible reasons will be surveyed.

### Further assessments

Optional saliva samples will be taken between t0 and t1 to assess cortisol awakening response and salivary alpha-amylase activity as objective *stress* markers. Salivary analyses will be conducted in the laboratory of the Institute of Medical Psychology and Behavioral Immunobiology, University Hospital Essen, Essen, Germany, as part of central scientific project Z02 within our CRC 289 Treatment Expectation. Brain imaging data on *functional and structural connectivity* will be collected on-site. Data will be contributed to central scientific project Z03 within our CRC 289 Treatment Expectation. Standardized MR protocols will be provided by Z03, who will use the data for pooled and meta-analytic analyses. Furthermore, we will include assessments of *past experiences with antidepressant use and discontinuation*, *well-being*, and *substance use*. As part of the CRC standard battery, we will measure *treatment expectations*, *personality traits*, *state and trait anxiety and depression*, *behavioral approach and avoidance tendencies*, *psychopathology*, *subjective impairment*, *perceived warmth and competence* of both study physician/psychologist and participant, *suspicions about treatment*, and extensive *demographic and medical characteristics*.

### Safety endpoints

Safety endpoints comprise depressive symptoms (BDI-II, MADRS) including inspection of suicidality and recurrence (SCID-5 CV; see above), current treatment effects, AEs including burdensome life events, and clinical impression according to psychopathological findings. A modified version of the *Generic Rating Scale for Treatment Effects* (GEEE_ACT_; 73) will be used to measure self-reported *current treatment effects*. Participants indicate worsening of their condition and treatment side effects (0 ‘*no worsening*’—10 ‘*greatest worsening imaginable*’, and 0 ‘*no complaints*’—10 ‘*greatest complaints imaginable*’, respectively) on an NRS with eleven response options (0–10) each. A score ≥ 8 on either item indicates severe distress. Occurrence of AEs will be assessed via interview, followed by an expert-rating of intensity and causal relation to study treatment. AEs will be graded with regard to i) intensity according to *Common Terminology Criteria for Adverse Events* (Grade 1 ‘*mild*’, Grade 2 ‘*moderate*’, Grade 3 ‘*severe*’, Grade 4 ‘*life threatening or disabling*’, Grade 5 ‘*fatal resulting in death*’; CTCAE; [78]), and ii) causality according to *World Health Organization—The Uppsala Monitoring Centre system* (1 ‘*certain*’, 2 ‘*probable/likely*’, 3 ‘*possible*’, 4 ‘*unlikely*’, 5 ‘*conditional/unclassified*’, 6 ‘*unassessable/unclassifiable*’; WHO-UMC; [79]).

### Statistical analysis

Analyses will be conducted per endpoint, i.e., as soon as all data of the according endpoint are available. A statistical analysis plan (SAP) will be established in collaboration with the Medical Biometry department of the University Medical Center Hamburg-Eppendorf, Hamburg, Germany, and published on ClinicalTrials.gov prior to analyses.

Analyses of the primary endpoint and of secondary endpoint discontinuation symptom load over the clinical observation phase will be based on the intention-to-treat population. For all other secondary endpoints, the full analysis set will be used. No interim analyses are planned.

Confirmatory testing of the interaction of treatment and treatment expectation in modulating discontinuation symptom load will be performed using a two-way ANCOVA. The model will contain the nominally scaled between-subject factors treatment (discontinuation vs. continuation) and treatment expectation (high vs. moderate), their interaction, the interval-scaled dependent variable discontinuation symptom load (measured as AUC) and, as covariates, the two binary stratification variables long-term use (yes vs. no) and risk of developing discontinuation symptoms (high vs. moderate) as well as discontinuation symptoms at baseline. Missing data of the primary outcome will be imputed using linear interpolation or, in case of individual termination of study treatment or rescue medication, as last observation carried forward.

If analyses reveal a significant interaction effect of treatment and treatment expectation, exploratory post-hoc comparisons will be made between experimental groups: A post-hoc comparison between the two continuation groups is planned to test whether participants show higher discontinuation symptom load with moderate (HC) than with high (OC) treatment expectations. Hence, conclusions could be drawn about a nocebo-induced effect of treatment expectation on discontinuation symptom load. To examine the extent to which discontinuation symptom load may be influenced by pharmacological factors alone, a post-hoc comparison will be performed between the moderate treatment expectation groups who discontinued (HD) versus remained on antidepressants (HC). A post-hoc comparison between participants who discontinued antidepressants will examine whether high treatment expectation (OD) is associated with higher discontinuation symptom load than moderate treatment expectation (HD).

We assume that the relationship between treatment expectation and discontinuation symptom load will vary according to stress ratings, antidepressant-related side effects, prior discontinuation experience, neuroticism, anxiety, somatosensory amplification, and illness framework. In case of a significant interaction within the primary analysis, individual moderators will be included as 3^rd^ factor in the model and three-way interactions will be analyzed.

Secondary endpoints will be analyzed as follows: Interactions of pharmacological and expectation effects in modulating i) discontinuation symptom load over the clinical observation phase measured as AUC, ii) stress, and iii) state anxiety will be examined following the model of our primary analysis. A binary logistic model will be used to predict iv) recurrence during the 13-week experimental period. A Cox proportional hazards model will be used to examine v) recurrence over the course of the complete trial. Finally, we will examine the interaction of expectation and emotion in modulating vi) attentional and emotional processing in a linear mixed model. This model will include a bias score (emotional versus neutral faces, measured in ms) as dependent variable and the factors *expectation concerning occurrence of discontinuation symptoms* (high (OD) vs. moderate (HC, HD) vs. none (OC)) and *emotion* (happy vs. sad vs. fearful), their 3 × 3 interaction, the stratification variables as covariates and random intercept for the individual patients. Analysis of secondary and safety endpoints and modulating factors will be explorative. Further specifications on statistical analyses can be found in the SAP.

Concerning analysis of the blood samples, we will check adherence to study medication by individually comparing antidepressant blood serum levels at t1 and t9. We will assess whether antidepressant blood serum levels lie within therapeutic range at t1. Finally, we will exploratively examine the relationship between initial antidepressant blood serum levels and discontinuation symptom load reported by the two discontinuation groups using simple correlation analyses.

### Data and safety monitoring

**Fig. 3:**
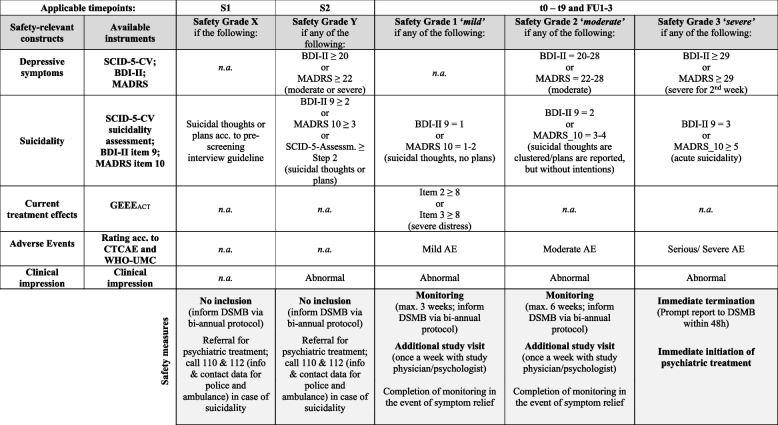
Schematic display of safety measures and procedures

Safety endpoints will be assessed and documented at every measurement point. Safety assessments and consequent actions follow a pre-defined, step-wise procedure (Fig. 3). Criteria for individual withdrawal from study treatment will be reviewed during each study visit and, in case of monitoring, during each additional visit. Treatment will be terminated in case of participant’s withdrawal of his/her informed consent, pregnancy, medical or psychological objections by the study physician or psychologist, or insufficient compliance regarding requirements for study participation including non-adherence. In these cases, the study physician informs about further treatment options and participants will be invited to continue participation in all measurement points. If a participant falls ill during the discontinuation process, a physician may decide to extend the discontinuation process by the duration of the illness (max. 4 weeks). If so, the participant will continue to receive the currently administered dose of medication from the study team. Measurements will be postponed accordingly. No additional interim measurements will take place. The continuation of the entire study will be questioned by the principal investigator in case of medical or psychological concerns/reoccurring AEs with possible causal relation to the study treatment regimen, insufficient study activity (e.g. enrolment rate < 5 per year), or unforeseeable complications that do not justify study continuation.

#### Data and safety monitoring board

An independent Data and Safety Monitoring Board (DSMB) will oversee recruitment and retention of participants. The DSMB will meet annually in order to monitor the study according to *Guidelines of Good Clinical Practice* [80]. The DSMB will receive bi-annual reports on progress of the study and anonymized safety-relevant participant data. Individual reports will be submitted within 48 h whenever a participant meets criteria for *Safety Grade 3* (Fig. 3).

#### Data management and pseudonymization

All data will be collected and handled in accordance with the European General Data Protection Regulation (GDPR; [81]). Data collection and analysis of psychometric measures will be performed by study staff at the University Medical Center Hamburg Eppendorf, Hamburg, Germany. Analysis and storage of saliva samples will take place the University Hospital Essen-Duisburg, Essen, Germany. Analysis and storage of the blood samples will take place at the Department of Legal Medicine, University Medical Center Hamburg Eppendorf, Hamburg, Germany. All psychometric and neuroendocrine measures will be made available to central scientific project Z02, all neuroimaging data will be contributed to central scientific project Z03 within our CRC 289 Treatment Expectation. Beyond that, no external bodies will be involved. To convert personal data into a pseudonym, we will use the software tool ALIIAS, which implements a dual authenticated, decentralized, encryption-based, deterministic pseudonymization technique [82]. Study results will preferably be shared via open-access publications and disseminated in lay language via outreach channels of CRC 289.

### Ethics, informed consent procedure and trial registration

This trial was approved by the ethics committee of the Hamburg Medical Chamber (reference number: PV7151, 16.12.2019). Research will be performed in accordance with the Declaration of Helsinki. All participants will provide written informed consent to participate. This trial has been registered at ClinicalTrials.gov (NCT05191277).

## Discussion

Our randomized, balanced, open-hidden trial is the first to explore the interplay of pharmacological effects and patients’ expectations in antidepressant discontinuation. To disentangle these effects, patients with fully remitted MDD will be randomly assigned to discontinue or remain on their antidepressant. Patients' expectations will be manipulated by varying verbal instructions using the open-hidden paradigm. Within the open trial arms, participants will receive full information about treatment, i.e., high expectation. Within the hidden trial arms, participants will be informed about a 50% chance of discontinuing versus remaining on their antidepressant, i.e., moderate expectation. Discontinuation symptom load will be assessed as primary outcome over the course of the 13-week experimental phase. Treatment (discontinuation vs. continuation) and treatment expectation (high vs. moderate) are expected to interact in modulating discontinuation symptom load.

Throughout our trial, we will closely monitor depressive symptoms to carefully distinguish discontinuation symptoms from recurrence. Thereby, we address the common bias in discontinuation trials of confounding recurrence with discontinuation symptoms [28]. We offer patients close supervision during discontinuation. We will regularly assess adverse events and adherence, apply an extensive psychometric battery, examine blood and saliva samples for antidepressant blood serum level and objective stress ratings, and measure functional and structural connectivity of the prefrontal cortex at rest. Hence, we aim to contribute to further research gaps in antidepressant discontinuation. Following patients up over a total period of one year will allow examining predictors of patients’ individual trajectories of discontinuation symptom load and recurrence [41–43].

The following limitations should be noted, however: First, we based our sample size calculations on medium to large effect sizes for expectation effects on depressive symptoms and treatment outcome [47, 50] and hypothesized small to medium interaction effects with pharmacological modulations. We decided to use this approximation because, to our knowledge, no study to date has explored the interplay of pharmacological and expectation effects in discontinuation of psychotropic drugs. Second, albeit German national guidelines effective at the time of study start recommended gradual dose-reduction over four weeks [26], van Leeuwen et al. (2021) concluded that tapering regimens up to four weeks do not reduce risk of discontinuation symptoms compared to abrupt discontinuation [28]. German treatment guidelines were recently updated and now advise tapering off antidepressants over a period of at least 8–12 weeks [83]. Four-week tapering regimens may be regarded as too rapid by patients [27], and impede recruitment. To ensure maximum patient safety, we considered associated risk of developing discontinuation symptoms for each antidepressant [37] and approximated hyperbolic tapering advocated by recent research [54]. Third, the intensive medical and psychological support provided to patients as part of our trial is not common practice, which could possibly mitigate negative expectation effects compared to discontinuation in the German health care system. However, we acknowledge that the recently updated German treatment guidelines now advise that treating physicians inform patients about duration of antidepressant use and possible difficulties arising from discontinuation early on and monitor patients closely throughout the discontinuation process [83]. As implementation of new guideline recommendations in routine clinical practice remains unsolved, we hope that our trial can contribute helpful insights that accelerate realization of the newly advised discontinuation strategies. We recommend that future research conducts discontinuation trials integrated within the national health care system, considers patients’ needs, closely collaborates with different professions in mental health (e.g., general practitioners, psychiatrists, pharmacists, psychotherapists), and applies extended, individualized tapering schemes.

Appropriate discontinuation of antidepressants is an unresolved clinical problem with serious negative implications for individual and society. Discontinuation symptoms are common and differential diagnosis with recurrence remains challenging, hindering indicated discontinuation. Negative expectations and nocebo effects are likely to play an important role in failed or neglected discontinuation attempts. In light of increasing unnecessary long-term use of antidepressants and the associated societal costs and individual burden, established rationales for safe and effective discontinuation are needed. We aim for a better understanding of mechanisms underlying discontinuation symptom load by disentangling pharmacological and expectation effects, while carefully monitoring recurrence. We hope to aid the development of interventions that support patients and physicians in discontinuing antidepressants more safely and effectively by targeting expectations towards discontinuation. Optimizing expectations before, during, and after discontinuation may prevent premature stopping of discontinuation attempts, positively influence the discontinuation process, and reduce discontinuation symptom load overall.

## Supplementary Information

**Additional file 1.** SPIRIT checklist.

**Additional file 2.** Summary of study registration in accordance with World Health Organization Trial Registration Data Set.

## Data Availability

Individual participant data will be shared with the study team of the CRC/TRR 289 after deidentification and will be available in this form for other researchers upon reasonable request. Only anonymized data in agglomerated form is used for publications. No personal data will be shared.

## Abbreviations

AE: adverse event
BAS Scale: Behavioral Approach System Scale of the BIS/BAS Questionnaire
BDI-II: Beck-Depression-Inventory II
BFI-10: Big Five Inventory-10
BIS Scale: Behavioral Inhibition System Scale of the BIS/BAS Questionnaire
CONSORT: Consolidated Standards of Reporting Trials
CRC: collaborative research center
CTCAE: Common Terminology Criteria for Adverse Events
DESS: Discontinuation Emergent Signs and Symptoms Scale
DFG: Deutsche Forschungsgemeinschaft (German Research Foundation)
DSMB: Data and Safety Monitoring Board
FU1-FU3: follow up 1 to 3
GASE: Generic Assessment of Side Effects
GDPR: European General Data Protection Regulation
GEEE: Generic Rating Scale for Previous Treatment Experiences, Treatment Expectations, and Treatment Effects
HC: hidden continuation
HD: hidden discontinuation
ITT: intention-to-treat
IVDR: In-vitro-Diagnostic Device Regulation
MADRS: Montgomery-Asberg Depression Rating Scale
ms: millisecond
MDD: Major Depressive Disorder
NaSSa: noradrenergic and specific serotonergic antidepressant
NRS: numeric rating scale
OC: open continuation
OD: open discontinuation
PDI: Pain Disability Index
PHEA: pharmacological and expectation effects in antidepressant discontinuation
PHQ-4: Patient Health Questionnaire-4
PSS-10: Perceived Stress Scale
rsfMRI: resting state functional magnetic resonance imaging
S1: first screening
S2: second screening and enrolment
SAP: statistical analysis plan
SCID-5-CV: Structured Clinical Interview for DSM-5 – Clinician Version
SOP: standard operating procedure
SSNRIs: selective serotonin-noradrenalin reuptake inhibitors
SSAS: Somatosensory Amplification Scale
SSRIs: selective serotonin reuptake inhibitors
STADI: State-Trait-Anxiety-Depression-Scale
SWEMWBS: Short Warwick-Edinburgh Mental Well-Being Scale
t0-t9: time of measurements
TEX-Q: Treatment Expectation Questionnaire
WHO: World Health Organization
WHO-UMC: World Health Organization -The Uppsala Monitoring Centre
Z02/Z03: central scientific projects of the collaborative research center
