# Supplementary material for: Disentangling pharmacological and expectation effects in antidepressant discontinuation among patients with fully remitted major depressive disorder: study protocol of a randomized, open-hidden discontinuation trial
Source: BMC Psychiatry. 2023 Jun 21;23:457. doi: 10.1186/s12888-023-04941-3 (PMC10286435; doi:10.1186/s12888-023-04941-3)
Supplement: Supplementary file 2 — Additional file 2. Summary of study registration in accordance with World Health Organization Trial Registration Data Set. [file 12888_2023_4941_MOESM2_ESM.docx]

Supplementary file 2. Summary of Registration according to WHO Trial Registration Data Set

|  | |  |
| --- | --- | --- |
| **Data category** | **Information** | |
| Primary registry and trial identifying number | ClinicalTrials.gov  NCT05191277 | |
| Date of registration in primary registry | January 13, 2022 | |
| Secondary identifying numbers | PV7151 CRC 289 Project A15 (Other Grant/Funding Number: DFG Deutsche Forschungsgemeinschaft ) U1111-1271-7231 (Other Identifier: Universal Trial Number) | |
| Source(s) of monetary or material support | This research is funded by the Deutsche Forschungsgemeinschaft (DFG, German Research Foundation): TRR 289 Treatment Expectation - Project Number 422744262. | |
| Primary sponsor | Universitätsklinikum Hamburg-Eppendorf (University Medical Centre Hamburg-Eppendorf; please contact [y.nestoriuc@hsu-hh.de](mailto:y.nestoriuc@hsu-hh.de) (Principal investigator) for any queries) | |
| Secondary sponsor(s) | n.a. | |
| Contact for public queries | YN, [y.nestoriuc@hsu-hh.de](mailto:y.nestoriuc@hsu-hh.de) (Principal investigator) | |
| Contact for scientific queries | YN, [y.nestoriuc@hsu-hh.de](mailto:y.nestoriuc@hsu-hh.de) (Principal investigator) | |
| Public title | PHEA study (Disentangling psychological and pharmacological effects in antidepressant discontinuation) | |
| Scientific title | Disentangling Pharmacological and Expectation Effects in Antidepressant Discontinuation | |
| Countries of recruitment | Germany | |
| Health condition(s) or problem(s) studied | Depressive Symptoms  Expectations  Antidepressants | |
| Intervention(s) | Drug: Treatment 'discontinuation of antidepressant medication'  Pharmacological intervention: Participants will discontinue their antidepressant medication.  Drug: Treatment 'continuation of antidepressant medication'  Pharmacological intervention: Participants will remain on their antidepressant medication.  Behavioral: Expectation 'high'  Psychological intervention: Participants' expectations will be manipulated by varying verbal instructions using the open-hidden paradigm. Within the open trial arms, participants will receive full information about their treatment (i.e., high expectation). expectation).  Behavioral: Expectation 'moderate'  Psychological intervention: Participants' expectations will be manipulated by varying verbal instructions using the open-hidden paradigm. Within the hidden trial arms, participants will be informed about a 50% chance of discontinuing versus remaining on their antidepressant medication (i.e., moderate expectation). | |
| Key inclusion and exclusion criteria | Key inclusion criteria   - Adult patients (18-75 years) with fully remitted MDD, single or recurrent, as main diagnosis. - Use of SSRI/SNRI or NaSSA. - Discontinuation wish by patient, supported by prescribing physician. - Fulfilment of guideline recommendations to discontinue antidepressant use.   Key exclusion criteria   - Acute or chronic somatic illness. - Acute suicidality. - Psychopathology that may interfere with study participation or medication adherence. - Insufficient German language proficiency. - No informed consent. | |
| Study type | Interventional  Allocation: Randomized Intervention Model: Parallel Assignment  Masking: Triple (Participant, Investigator, Outcomes Assessor)  Primary Purpose: Basic Science | |
| Date of first enrolment | 15.09.2022 | |
| Target sample size | 196 | |
| Recruitment status | Recruiting | |
| Primary outcome(s) | Discontinuation symptom load over the course of the experimental phase (12 weeks) assessed as Area under the Curve (adjusted for pseudo-baseline) | |
| Key secondary outcomes | Secondary outcomes will include i) discontinuation symptom load over the clinical observation period, ii) recurrence over the experimental period, iii) recurrence over the course of the complete trial evaluated in a time-to-event analysis, differences in iv) stress and v) state anxiety from baseline to end of the experimental phase, and vi) attentional and emotional processing at the end of the experimental phase. | |
| Version protocol | Version 1: September 12^th^, 2019 (as submitted to ethics committee)  Version 2: October 29^th^, 2019 (resubmitted with minor changes for fulfilment of requirements relating to initial submission)  Version 3: March 30^th^, 2021 (1^st^ amendment to add a pilot study and change public study tile)  Version 4: July 15^th^, 2021 (2^nd^ amendment to add optional rsfMRI measurement)  Version 5: May 18^th^. 2023 (3^rd^ amendment to add changes following piloting the study (e.g., primary and secondary outcomes, safety endpoints, data and safety monitoring, statistical analyses) and resubmission with all prior changes tracked and summarized) | |
